# Visual attention and postural stability among older adults participating in health-enhancing physical activity: a systematic review

**DOI:** 10.3389/fnetp.2026.1841735

**Published:** 2026-06-08

**Authors:** Michael Joseph Dino, Chloe Margalaux Villafuerte, Jerald Sayat, Justin Pimentel, Jelaine Beniopa, Princess Sarah Bahaynon, Abraham John Cuevas, Ma. Lucita Alonzo, Gerald Dino, Janet Lopez, Ladda Thiamwong, Mona Shattell

**Affiliations:** 1 College of Nursing, University of Central Florida, Orlando, FL, United States; 2 Research Development and Innovation Center, Our Lady of Fatima University, Valenzuela, Philippines; 3 College of Medicine, Our Lady of Fatima University, Valenzuela, Philippines; 4 The Libraries, De La Salle University, Manila, Philippines

**Keywords:** eye-tracking, older adults, postural stability, systematic review, visual attention

## Abstract

**Introduction:**

As the global population ages, declines in sensory, cognitive, and motor functions increase fall risk and compromise postural stability. Despite growing research, systematic reviews examining visual attention and postural stability—particularly through eye-tracking—remain scarce. Grounded in a network physiology perspective, this review aimed to (1) describe bibliometric characteristics, (2) thematize study purposes and outcomes, (3) map conceptual linkages through keyword network analysis, and (4) generate insights into the relationship between visual attention and postural stability in older adults during physical exercise interventions.

**Methods:**

Following a five-stage integrative review process using Covidence, 15 studies were included in the final analysis.

**Results:**

Bibliometric findings show that publications were multi-authored (93.33%), appeared in health-related journals (100%), peaked in 2022 (20.00%), and originated predominantly from the Americas (46.67%). Most studies focused on diagnosis (26.67%), involved healthy participants (46.67%), and used small samples (Md = 30; IQR = 28) in laboratory or clinical settings. Thematic analysis yielded five domains—Performance, Program, Process, Product, and Person (5Ps)—while keyword network analysis identified seven clusters: Vision, Virtual, Vulnerability, Visual-Motor, Velocity, Vestibular, and Vergence, collectively emphasizing a multifaceted, systems-oriented approach to fall prevention. Among visual attention metrics, fixation (21.15%) and saccades (19.23%) were most frequently assessed, and both were consistently associated with posture, balance, gait, and stability outcomes. Advanced metrics such as heat maps, pupil dilation, dwell time, and re-fixation remain underutilized.

**Discussion:**

Overall, this review establishes visual attention as a central and modifiable determinant of postural stability, with gaze-based, technology-assisted, multicomponent approaches warranting priority in fall prevention assessment and intervention.

**Systematic Review Registration:**

https://osf.io/bmsw6/overview.

## 1 Introduction

The world is entering an era of rapid population aging, with the number of individuals aged 60 and older rising from one billion in 2019 to 1.4 billion by 2030 and 2.1 billion by 2050. This surge is happening at an unprecedented rate and is expected to accelerate further globally (World Health Organization, 2026). The aging population will significantly impact economies, social services, healthcare systems, communities, and family structures. This profound demographic shift also brings complex and evolving health risks (Khan et al., 2024), including chronic diseases, sensory decline, and reduced mobility, all of which can compromise their independence and quality of life (Guo et al., 2022). Given these realities, there is an urgent and growing need to prioritize the health and wellbeing of older adults (Abdi et al., 2019). Addressing the complex, multifaceted health challenges of this population requires targeted research, evidence-based interventions, and health policies that are responsive to the unique physiological and functional needs of aging individuals (Theodorakis et al., 2025). Neglecting this issue may further intensify the social and economic burdens already associated with the rapidly growing older population (Tang et al., 2022).

Among the many health concerns facing older adults, postural stability is a critical yet often overlooked concept (Wang et al., 2022). Postural stability refers to an individual’s ability to maintain the body’s center of mass within the base of support under both static and dynamic conditions (Kenis-Coskun et al., 2016). Alarmingly, falls are a direct consequence of compromised postural stability (Gandawidura and Ikeda, 2025) and are the leading cause of injury-related morbidity and mortality among older adults, affecting one in three individuals aged 65 and older annually, with approximately 10% experiencing multiple falls each year (Centers for Disease Control and Prevention, 2026). These falls are frequently serious in nature, carrying significant risks of morbidity and mortality while threatening the functional independence of older individuals (Vaishya and Vaish, 2020). Maintaining adequate postural stability is therefore essential not only for preventing falls and their associated complications, but also for preserving functional independence, mobility, and overall quality of life in the aging population (Montero-Odasso et al., 2022).

Research on postural stability in older adults has explored a wide range of contributing factors, such as muscle strength (Wang et al., 2022), proprioceptive sensitivity (Liu et al., 2023), vestibular function (Agrawal et al., 2020), and cognitive performance during dual-task (Vermette et al., 2023). Collectively, these findings highlight postural stability as a dynamic, cognitively driven process rather than a purely biomechanical one. This is further supported by the frameworks of network physiology, which state that postural control emerges from the dynamic interactions among multiple physiological systems such as neural, sensory, and motor networks, rather than the isolated function of individual components (Bartsch et al., 2015; Ivanov et al., 2017). These foundational frameworks demonstrate that physiological systems continuously exchange information through time-varying networks, where stability is maintained through coordinated inter-system connectivity. Among these factors, visual attention appears to be one of the most significant components of postural stability. As defined (McMains and Kastner, 2009), visual attention is a set of cognitive mechanisms by which the brain prioritizes relevant visual information while filtering out distractions, especially in complex visual environments. Considering the age-related decline of sensory and cognitive functions in older adults (Abbas et al., 2024), a thorough understanding of the role of visual attention in mediating postural stability is essential for informing assessment and intervention strategies.

While research in this area has progressed considerably, previous reviews have largely examined visual attention and postural stability separately, with emphasis on either cognitive aspects of balance control or general postural outcomes, and limited integration of eye-tracking evidence (Walsh and Snowball, 2023). Most syntheses have also been descriptive in nature, with few attempts to systematically map how specific visual attention metrics relate to postural stability across different study contexts (Rossiter et al., 2017). Moreover, the literature lacks a unified systems-level approach through network physiology to explain how these variables interact (Yeh et al., 2014), limiting understanding of their integrated function. Existing reviews have also rarely applied bibliometric or network-based methods to identify research trends, thematic structures, and conceptual gaps. This lack of integrative synthesis constrains both theory and practice, particularly in older adults, where declining attentional control affects the integration of visual, proprioceptive, and vestibular inputs, increasing fall risk (Ma et al., 2022). In light of these gaps, this systematic review is guided by the question: Based on the current body of literature, how is visual attention (eye-tracking metrics) linked to postural stability in older adults? It aims to synthesize evidence using a network physiology perspective, specifically by (1) describing bibliometric characteristics of relevant studies, (2) thematizing study purposes and outcomes, (3) conducting keyword network analysis to map conceptual linkages, and (4) generating insights on visual attention–postural stability interactions, ultimately contributing to a more integrated understanding of postural control in older adults.

## 2 Methods

This review adopted a hybrid methodological approach that integrates the rigor of a systematic review with the inclusive nature of an integrative review. Guided by the integrative review framework (Whittemore and Knafl, 2005), the study followed a structured five-phase process consisting of (a) problem identification, (b) comprehensive literature search, (c) data evaluation, (d) data analysis, and (e) presentation. Moreover, the Preferred Reporting Items for Systematic Reviews and Meta-Analyses (PRISMA) guidelines (Moher et al., 2010) were incorporated into the reporting of results to uphold quality and consistency throughout the review process. This study received ethics clearance exemption and has been registered at OSF.io (embargoed).

### 2.1 Stage 1: problem identification

Problem identification guides the formulation of relevant research questions prior to further analysis. This paper investigates the current state of research on visual attention and postural stability in older adults, a pressing concern given the significant and ongoing growth of the global aging population. Despite its clinical relevance, this field remains fragmented and lacks a comprehensive synthesis, as most studies examine visual attention and postural stability independently, with limited integration of eye-tracking technology, constraining both theoretical and practical understanding of how declining attentional control contributes to fall risk in this population. Hence, a systematic review of existing literature is essential to develop a comprehensive understanding of its current scope, methodological approaches, and existing gaps. Accordingly, this review is guided by the following inquiries: (1) What are the bibliometric characteristics and publication trends of studies in this field? (2) What themes describe the overall research purposes and outcomes? (3) What conceptual clusters are evident from keyword network analysis? and (4) How is the relationship between visual attention and postural stability characterized across studies?

### 2.2 Stage 2: literature search strings

Table 1 displays the database search strategy utilized to identify the research articles reviewed in this paper. Specifically, databases including Scopus, PubMed, and Web of Science were searched for relevant studies using keywords such as visual attention, postural stability, eye-tracking, physical activity, and older adults.

**TABLE 1:** Literature search string.

Database	Search string	Results
Scopus	TITLE-ABS-KEY ((exercise OR training OR “physical activity” OR “exercise intervention”) AND (“eye tracking” OR “visual attention” OR “gaze” OR “oculomotor”) AND (“postural control” OR “postural stability” OR balance OR “postural sway”) AND (“older adults” OR elderly OR aging OR geriatric)) AND (LIMIT-TO (LANGUAGE, “English”))	66
PubMed	(exercise OR training OR “physical activity” OR “exercise intervention”) AND (“eye tracking” OR “visual attention” OR “gaze” OR “oculomotor”) AND (“postural control” OR “postural stability” OR balance OR “postural sway”) AND (“older adults” OR elderly OR aging OR geriatric)	162
Web of Science	TS=(exercise OR training OR “physical activity” OR “exercise intervention”) AND TS=(“eye tracking” OR “visual attention” OR “gaze” OR “oculomotor”) AND TS=(“postural control” OR “postural stability” OR balance OR “postural sway”) AND TS=(“older adults” OR elderly OR aging OR geriatric) AND LA = English	102
​	Total	330

### 2.3 Stage 3: data evaluation

Article screening was facilitated by Covidence, a web-based software platform specifically for review management, to support the validation and evaluation of the retrieved articles. Once the articles were imported into Covidence, duplicate entries were automatically detected and removed by the software or manually by the reviewers. Initial screening, including title and abstract review, was performed by at least two independent reviewers, followed by full-text screening of the remaining articles. Inter-rater agreement for study screening was recorded (Cohen’s κ = 0.84), while percent agreement for data extraction reached 92%, reflecting relatively strong consistency between reviewers. Any conflicts arising from disagreements among reviewers were resolved by the principal investigator. To determine the eligibility and relevance of the full-text articles, the following inclusion criteria were considered through the PICOS framework, as presented in Table 2.

**TABLE 2:** Eligibility criteria based on the PICOS framework.

PICOS	Inclusion criteria	Exclusion criteria
Population	60 years old and above	Individuals aged 59 years and below
Intervention	Hardware/software forms of Eye-tracking technology and postural stability technology	Non-postural or non-visual assessment tools
Comparision	Not applicable as the systematic review focuses on older adults only	Comparison between older and younger adults
Outcomes	Visual attention metrics; postural stability indicators (falls, balance, stability, and gait); multiple body systems (visual, vestibular; proprioceptive systems)	No reports of both visual attention and postural stability variables
Study design	Empirical and theoretical research	Review papers; conference papers; and non-peer-reviewed white papers

The PICOS framework established a focus scope of the systematic review by through its inclusion and exclusion criteria specified through the Population, Intervention, Comparison, Outcomes, and Study Design. These criteria are further elaborated below:

*Population and Outcomes:* This review selected studies that examine the relationship between visual attention and postural stability in older adults aged 60 years and older. Given that aging places this population at elevated risk for sensory and motor dysfunction that compromises functional independence and increases fall risk, studies must include variables reporting postural stability, encompassing falls, balance, stability, and gait, alongside visual attention metrics. Studies that examine multiple body systems, such as the visual, vestibular, and proprioceptive systems, are likewise included to reflect the integrative and multisystem nature of postural control.

*Intervention:* This review included studies that used eye-tracking technology to capture visual attention metrics. Eye-tracking provides objective, quantifiable data on gaze patterns and attentional processes (Patarini et al., 2025), making it an essential tool for examining how visual attention operates under static and dynamic postural conditions. Furthermore, postural stability assessments that measure falls, balance, stability, and gait were likewise considered eligible, as they provide direct, measurable indicators of an individual’s ability to maintain postural control. Both hardware- and software-based implementations of these assessment tools for physical activities in older adults are included in this review.

*Study design:* This review included empirical and theoretical research retrieved from various databases. Conference papers and review papers were excluded to ensure that only primary empirical evidence is synthesized, as this practice is consistent with standard systematic review methodology (Page et al., 2021) and upholds the methodological rigor and validity of the findings.

*Study language:* This study selected articles written in or translated into English only. English is the primary language of global scientific discourse, with the vast majority of peer-reviewed journals disseminating research in English; therefore, restricting the review to English-language articles ensures alignment with international scientific standards and optimal study visibility.

The inclusion and exclusion criteria were deliberately established to encompass studies that concurrently investigates visual attention and postural stability in older individuals. This has implications, as it inherently limits the number of eligible studies, given that most of the literature investigates these variables independently. While broader criteria could have increased the number of included studies, this focused selection was necessary to maintain alignment with the study’s objective of exploring their interaction within a network physiology framework.

### 2.4 Stage 4: data analysis

For data analysis, the selected studies were independently examined by multiple researchers to identify recurring patterns and themes across the entire dataset. Four main categories of information were retrieved during the data extraction process: (1) article Bibliometrics, encompassing author, year of publication, bibliographic identifiers, article type, authorship, journal type, country of origin, and region (based from the World Health Organization); (2) critical Appraisal, including Mixed Methods Appraisal Tool 2018 (MMAT 2018) scores and relevance assessments; (3) article Focus, such as health focus, participant status, sample size, research settings, article purpose, and article outcomes; and (4) technology-based tools used to track and measure ten (10) visual attention metrics, as well as technology-driven tools and conventional clinical tests used to assess various parameters of postural stability among older adults.

To ensure consistency and transparency, reviewer disagreements during the screening and extraction phases were systematically addressed through the Covidence platform. The resulting extracted data were subsequently consolidated into a standardized spreadsheet and securely archived within institutional repositories for safekeeping. A more detailed summary of the data extraction spreadsheet is available in the Supplementary Material section.

### 2.5 Stage 5: search strategy limitations and sensitivity analysis

The search strategy was conducted with predefined methodological constraints to ensure transparency, reproducibility, and feasibility in accordance with PRISMA reporting standards. The search was limited to Scopus, PubMed, and Web of Science, selected based on their broad interdisciplinary coverage and established use in systematic reviews. While these databases index a large proportion of peer-reviewed biomedical and health sciences literature, relevant studies indexed exclusively in discipline-specific databases: CINAHL; PsycINFO; IEEE Xplore, may not have been retrieved.

Search terms were developed using broad conceptual constructs; “eye tracking,” “visual attention,” and “oculomotor” to account for heterogeneity in terminology across disciplines. However, more granular descriptors; “saccade,” “fixation,” “scanpath,” were not explicitly included, which may have reduced sensitivity for studies reporting eye-movement outcomes using metric-specific terminology.

In addition, eligibility was restricted to peer-reviewed journal articles to ensure inclusion of studies that had undergone formal methodological peer review. Grey literature sources, including conference proceedings, dissertations, and technical reports, were excluded. Only studies published in English were included to ensure consistency in screening and data extraction. These restrictions may have introduced publication and language bias.

A sensitivity analysis was conducted through manual screening of reference lists of included studies and relevant review articles to identify additional eligible records not captured in the database search. This process did not identify additional studies meeting the inclusion criteria; however, the possibility of missed evidence cannot be excluded. These methodological constraints reflect deliberate trade-offs made during review design and should be considered when interpreting the completeness of the evidence base.

Due to these methodological limitations and the intricacy of the inclusion criteria, only a restricted number of research fulfilled all qualifying requirements. This highlights the emerging and fragmented state of research on the relationship between visual attention and postural stability.

### 2.6 Stage 6: presentation

The findings from the review of related research articles were organized and presented in thematic tables, conceptual models, and figures for better visualization. This approach enabled systematic recognition and synthesis of common patterns, relationships among variables, overlooked gaps, and emerging trends within the body of literature on the network physiology of exercise, with particular focus on visual attention and postural stability in older adults.

**FIGURE 1:**
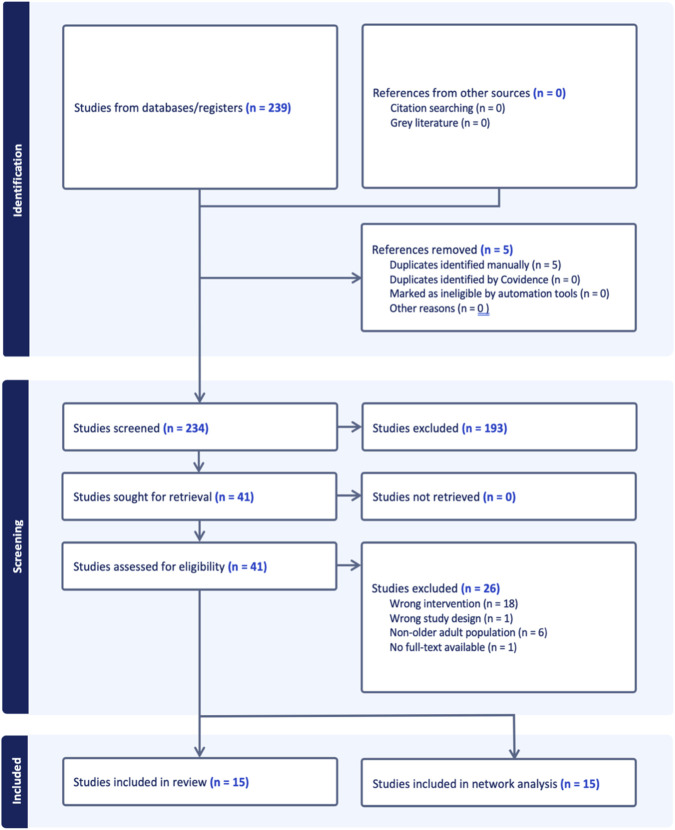
PRISMA diagram.

Figure 1 presents the PRISMA diagram illustrating the systematic process of identification, screening, and inclusion employed in this review. Of the 239 studies initially retrieved from databases, five duplicates were manually removed, leaving 234 studies for screening. Title and abstract review led to the exclusion of 193 studies that did not meet the established inclusion criteria. The remaining 41 studies underwent full-text assessment, during which 26 were further excluded due to the wrong intervention (n = 18), the wrong study design (n = 1), a non-older adult population (n = 6), or unavailability of the full text (n = 1). Ultimately, 15 studies met all eligibility requirements and were included in the final analysis, which likewise underwent keyword network analysis using VOSviewer version 1.6.20.

## 3 Results

### 3.1 Article bibliometrics

Table 3 showcases the bibliometric characteristics of the fifteen (n = 15) reviewed articles. Most studies were authored by multiple contributors (n = 14; 93.33%), and all were published as research articles in health-related journals (n = 15; 100%). In terms of publication year, 2022 had the highest number of studies (n = 3; 20.00%), followed by 2025 (n = 2; 13.33%), with the remaining years each contributing one study. The majority of studies originated from the Americas (n = 7; 46.67%), with additional contributions from the Eastern Mediterranean (n = 4; 26.67%), Europe (n = 3; 20.00%), and Southeast Asia (n = 1; 6.67%). Most studies focused on diagnosis (n = 4; 26.67%), followed by prevention and treatment (n = 3; 20.00 each), and primarily involved healthy participants (n = 7; 46.67%). Sample sizes were generally small, with most studies including 0–50 participants (Md = 30; IQR = 28). Finally, studies were commonly conducted in laboratory and clinical settings (n = 5; 33.33 each), with fewer in other settings.

**TABLE 3:** Article Bibliometrics.

Article attributes	f	%
Authorship		
Single	0	0.00
Double	1	6.67
Multiple	14	93.33
Journal Publication		
Health	15	100.00
Non-Health	0	0.00
Article Type		
Research	15	100.00
Theoretical	0	0.00
Year		
2008	1	6.67
2010	1	6.67
2013	1	6.67
2014	1	6.67
2016	1	6.67
2017	1	6.67
2018	1	6.67
2019	1	6.67
2021	1	6.67
2022	3	20.00
2023	1	6.67
2025	2	13.33
WHO Region		
Americas	7	46.67
Southeast Asian	1	6.67
European	3	20.00
East Mediterranean	4	26.67
Health Focus		
Promotion - Healthy	2	13.33
Prevention - At risk	3	20.00
Diagnosis - To detect	4	26.67
Treatment - Intervention	3	20.00
Rehabilitation - Recovery	1	6.67
General	2	13.33
Client Status		
Healthy	7	46.67
Non-Healthy	5	33.33
Both	3	20.00
Sample Size (Med = 30; IQR = 28)		
0–50	12	80.00
51–100	2	13.33
251–300	1	6.67
Research Environment		
Home	1	6.67
Fitness Facility	1	6.67
Laboratory	5	33.33
Community	1	6.67
Clinical	5	33.33
Simulation Room	1	6.67
Not Mentioned	1	6.67

Some sub-attributes with zero values are omitted.

#### 3.1.1 Quality of included studies

Table 4 presented the 15 articles included in this review, which examined the relationship between visual attention and postural stability among older adults, along with the eye-tracking technologies involved, were subjected to quality appraisal using the MMAT. Of the 15 studies, seven (46.67%) achieved a perfect score of 100%, suggesting high methodological quality, seven (46.67%) received a score of 80%, reflecting moderately high quality, and one (6.67%) received a score of 60%, which is acceptable but comparatively lower methodological rigor. These quality ratings suggest that the overall body of evidence synthesized in this review is scientifically credible, though one study rated at 60% should be interpreted with relative caution, as its findings may carry less evidentiary weight compared to the higher-rated studies. A further detailed breakdown of the MMAT scores by question per category for all included studies is provided in the Supplementary Material.

**TABLE 4:** Methodological quality assessment.

No.	Research	MMAT	RB	Purpose	Outcomes
1	Abasi et al. (2022)	60%	3	To examine the effects of vestibular rehabilitation on oculomotor function	Vestibular rehabilitation is underutilized but can improve balance and address impairments in individuals with neurological conditions
2	Alghamdi et al. (2021)	80%	4	To investigate the relationship between visual attention, balance, mobility, and performance in Kinect games	Visual attention measures are linked to performance in Kinect games and are also associated with balance and gait abilities
3	Chen et al. (2025)	80%	5	To examine how short-term interactive balance training influences cortical reorganization in older adults	Short-term visual-guided postural training improved stability in older adults, alongside enhanced brain activity and network organization
4	Cinelli et al. (2008)	60%	4	To compare balance and body segment control between older and younger adults during gaze reorientation tasks	Older adults showed delayed coordination between eye movements and body rotation, indicating age-related physiological decline
5	De Souza et al. (2022)	80%	5	To examine the eye–posture relationship in older fallers, focusing on visual stabilization mechanisms	Older adults with a history of fall had poorer balance and eye movements than nonfallers, though their eye–posture relationship remained functional
6	Fatima et al. (2022)	80%	4	To compare balance training with and without gaze stabilization exercises in elderly patients with chronic dizziness	Combining gaze stability and balance exercises effectively improves balance and reduces fall risk in older adults with dizziness
7	Maldonado-Díaz et al. (2025)	80%	5	To investigate visual attention using eye-tracking during VR-based balance training in older adults with cognitive impairment	Eye-tracking provides useful insights into attentional behavior during balance training and supports VR-based rehabilitation approaches
8	Matheron et al. (2016)	100%	5	To assess the effects of vision, vergence, viewing distance, and cognitive load on postural control in older adults	Postural stability decreased with far focus and eye closure but improved with vergence, highlighting proximity-related benefits in older adults
9	Mitsutake et al. (2018)	100%	5	To investigate the effects of posterior circulation stroke (PCS) on postural stability and evaluate gaze stability exercises in PCS and non-PCS groups	Gaze stability exercises improve postural control in PCS patients, particularly during dynamic standing tasks
10	Rodrigues et al. (2023)	100%	5	To examine postural control in diabetic older women during fixation and horizontal eye movements	The diabetic group showed poorer postural control, reflected by increased sway amplitude and velocity
11	Szturm et al. (2015)	80%	5	To evaluate the reliability and validity of a dual-task platform for assessing balance, gaze, and cognition in older adults	The platform showed moderate to high reliability and consistently detected changes in balance, gaze, and cognition under dual-task conditions
12	Tuunaeinen et al. (2011)	100%	5	To examine the relationship between vestibular symptoms and objective vestibular, oculomotor, and balance measures in older adults	Most elderly participants exhibited vestibular and balance impairments, with reduced postural control and multiple causes of dizziness and falls
13	Yamada et al. (2013)	100%	5	To assess the effects of a multitarget stepping program with multicomponent exercise on stepping, gaze, and fall outcomes	A twice-weekly multitarget stepping program combined with multicomponent exercise reduced falls and fractures while improving stepping, gaze, and physical performance over 12 months
14	Althomali et al. (2019)	100%	5	To determine if visual attention training can improve balance and mobility among older adults	Visual attention training showed no improvements in balance or mobility among older adults
15	Althomali and Leat (2018)	100%	5	To determine the relationship of balance, mobility, and fear of falling, and aspect of vision	Multiple visual factors contribute to impaired balance and mobility beyond what standard vision tests capture

MMAT, Mixed Methods Appraisal Tool (MMAT) Score: 0%–100% (0 - poor; 100 - excellent).

R^B^, Relevance: 0–5 (0 - not relevant; 5 - highly relevant).

### 3.2 Thematized purpose and outcome

**FIGURE 2:**
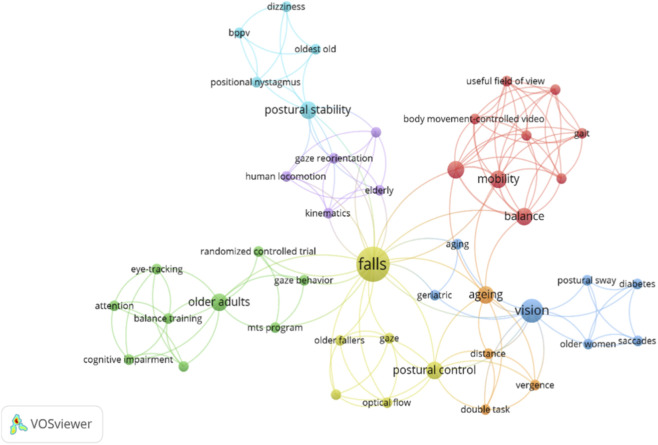
Article keywords network map.

Table 5 summarizes the included studies and reveals five key domains: “Performance”, “Program”, “Process”, “Product”, and “Person”, collectively termed the “5Ps of Visual Attention and Postural Stability.” Under “Performance”, findings showed that vision and eye movements play a critical role in postural stability, with oculomotor function directly influencing balance outcomes. In terms of “Program”, interventions integrating balance, gaze, and rehabilitation, particularly technology-assisted approaches, were effective in improving balance and reducing fall risk. The “Process” domain highlighted the importance of cognitive factors, especially attention, demonstrating strong associations with balance performance, particularly in dual-task conditions. For “Product”, standardized assessment tools reliably capture the integration of balance, gaze, and cognitive functions, supporting their utility for evaluating complex performance outcomes. Lastly, the “Person” domain highlighted variability across age groups and clinical populations, with aging and health conditions associated with declines in postural stability and visual attention. The 5Ps of Visual Attention and Postural Stability are also depicted in Figure 2 as a honeycomb structure, illustrating the connections and interdisciplinarity among the themes. This model highlights how each component reinforces the others, forming a cohesive system in which no single element operates in isolation. Together, they contribute to a more integrated and adaptive framework for optimizing both visual processing and postural control. A more detailed summary of the articles’ purposes and outcomes can be found in the Supplementary Material section.

**TABLE 5:** Thematized clusters of article keyword network map.

​	Purpose		Outcomes	
Theme	Description	Studies	Description	Studies
Performance	To examine vision and eye movement effects on balance	(Abasi et al., 2022; Althomali and Leat, 2018; Cinelli et al., 2008; Tuunainen et al., 2011; Matheron et al., 2016; Rodrigues et al., 2023; Souza Silva et al., 2019)	Vision and eye movements affect postural stability	(Althomali and Leat, 2018; Chen et al., 2025; Tuunainen et al., 2011; Matheron et al., 2016; Rodrigues et al., 2023; Souza Silva et al., 2019)
Program	To evaluate balance, gaze, and rehabilitation programs	(Abasi et al., 2022; Fatima et al., 2022; Mitsutake et al., 2018; Yamada et al., 2013)	Technology interventions improve balance and reduce falls	(Abasi et al., 2022; Fatima et al., 2022; Mitsutake et al., 2018; Yamada et al., 2013)
Process	To explore attention and cognition in balance tasks	(Alghamdi et al., 2021; Althomali and Leat, 2018; Chen et al., 2025; Maldonado-Díaz et al., 2025; Szturm et al., 2015)	Cognition is associated with balance performance variables	(Alghamdi et al., 2021; Maldonado-Díaz et al., 2025; Szturm et al., 2015)
Product	To assess tools measuring balance–gaze–cognition	(Tuunainen et al., 2011; Szturm et al., 2015)	Standardized tools effectively measured integrated functions	(Maldonado-Díaz et al., 2025; Szturm et al., 2015)
Person	To compare balance and visual attention across age and clinical groups	(Cinelli et al., 2008; Tuunainen et al., 2011; Mitsutake et al., 2018; Rodrigues et al., 2023)	Aging/conditions reduce postural stability	(Cinelli et al., 2008; Tuunainen et al., 2011; Mitsutake et al., 2018; Rodrigues et al., 2023; Souza Silva et al., 2019)

### 3.3 Article keyword network map

Figure 2 highlights the network map of article keywords that reveals seven (7) thematic clusters (Table 6) in the systematic review, summarizing the characteristics of existing studies on visual attention and postural stability among older adults. The network conveys that some of the most frequently occurring keywords among the 15 included papers consist of the keywords such as “falls”, “postural stability”, “older adults”, “balance”, “ageing”, and “vision” as represented by node sizes that correspond to word frequency and line weights for co-occurrence strength. Seven keyword clusters, specifically “Vision”, “Virtual”, “Vulnerability”, “Visual-Motor”, “Velocity”, “Vestibular”, and “Vergence”. The first cluster comprises studies on video game–based platforms designed to train visual attention and mobility in older adults. The second cluster centers on virtual reality–based interventions aimed at rehabilitating functional impairments in aging populations. The third cluster highlights keywords related to aging and associated comorbidities, particularly among older women. The fourth cluster focuses on optical flow and its role in postural control. The fifth cluster pertains to gaze reorientation and visuomotor coordination during locomotion in older adults. The sixth cluster is associated with vestibular disorders and their contribution to postural instability in advanced age. Finally, the seventh cluster includes keywords describing impairments in perception and coordination under dual-task conditions across varying distances.

**TABLE 6:** Thematized clusters of article keyword network map.

Cluster*	Keywords	Theme	Description
1	Balance, Body Movement-Control, Gait, Microsoft™ Xbox® 360, Mobility, Multiple Object Tracking, Useful Field of View, Video Games, Visual Attention	Vision	Study keywords related to video gaming platforms train visual attention, gaze, balance, and mobility in older adults
2	Attention, Balance Training, Cognitive Impairment, Eye-Tracking, Gaze Behavior, MTS Program, Older Adults, Randomized Controlled Trial, Virtual Reality	Virtual	Study keywords related to virtual reality and eye-tracking programs that rehabilitate cognitive and balance impairments in aging populations
3	Aging, Diabetes, Geriatric, Older Women, Postural Sway, Saccades, Vision	Vulnerability	Study keywords related to aging, diabetes, and vision decline reduce postural sway and stability in older women
4	Falls, Gaze, Older Fallers, Optical Flow, Postural Control, Visual-Motor Coupling	Visual-Motor	Study keywords related to gaze and optical flow regulate postural control and fall prevention in older fallers
5	Body Segments, Elderly, Gaze Reorientation, Human Locomotion, Kinematics	Velocity	Study keywords related to gaze reorientation and body segment kinematics coordinate movement during locomotion in the elderly
6	BPPV, Dizziness, Oldest Old, Positional Nystagmus, Postural Stability	Vestibular	Study keywords related to vestibular disorders such as BPPV and dizziness destabilize posture in the oldest old
7	Ageing, Distance, Double Task, Vergence	Vergence	Study keywords related to aging that impairs depth perception and gaze coordination during dual-task conditions at varying distances

Colors were matched with network keyword clusters.

### 3.4 Technology-driven assessment tools and metrics measuring various attributes of visual attention and postural stability

**FIGURE 3:**
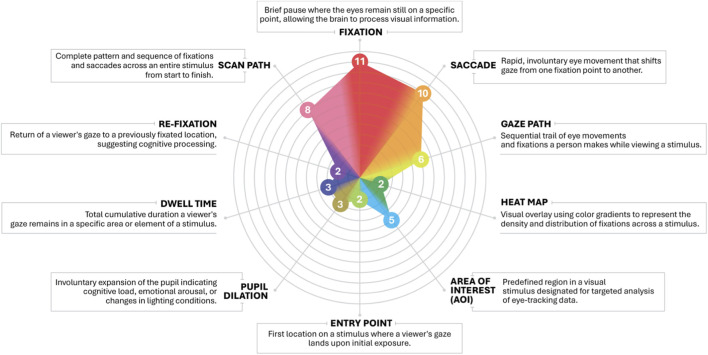
Radar chart of technologies measuring visual attention metrics.

The distribution of studies examining visual attention and postural stability attributes is presented using a radar map, with numbers corresponding to the number of studies that focus on the listed metric. As illustrated in Figure 3, the most frequently assessed visual attention metrics include Scan Path *(n = 8; 15.38%*), Fixation (*n = 11; 21.15%*), Saccade *(n = 10; 19.23%*), Gaze Path (*n = 6; 11.53%*) with fewer studies measuring Heat Map *(n = 2; 3.84%)*, Area of Interest *(n = 5; 9.61%)*, Entry Point (*n = 2; 3.84%*), Pupil Dilation (*n = 3; 5.76%)*, Dwell Time (*n = 3; 5.76%*), Re-Fixation (*n = 2; 3.84%*).

**FIGURE 4:**
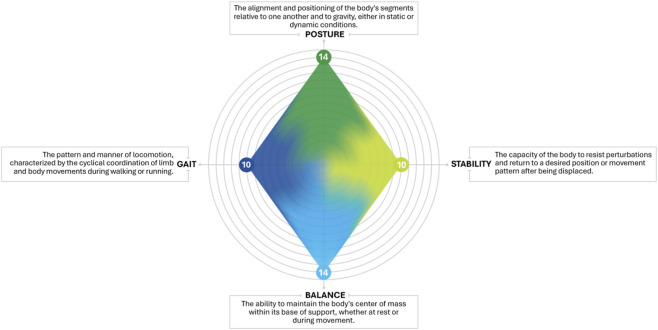
Radar chart of technologies measuring postural stability metrics.

Postural stability metrics are summarized in Figure 4. Measurements of Posture and Balance appear the most (*n = 14; 29.16% each*), and Gait and Stability (*n = 10; 20.83% each*).

### 3.5 Visual attention and postural stability measurements in published studies (radar map combined)

**FIGURE 5:**
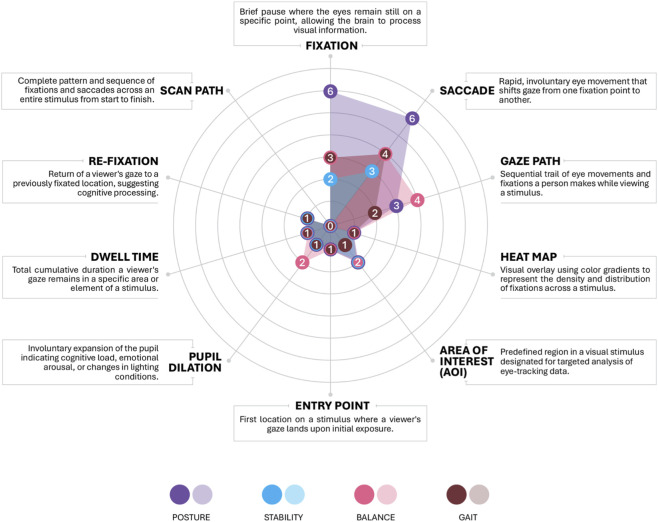
Radar chart of combined visual attention and postural stability metrics.

Figure 5 is a radar chart presenting a combined overlay of visual attention metrics by frequency of use across postural stability measures. In relation to posture, Fixation and Saccades were the most frequently used metrics (*n = 6; 27.27%, each*), followed by gaze (*n = 3; 13.63%*) and area of interest (*n = 2; 9%*). Heat maps, entry point, pupil dilation, dwell time, and re-fixation were each reported once (*n = 1; 4.5%*), while scan path was not used or reported (*n= 0)*. For Stability, Saccade was most frequently reported (*n = 3; 25%*) followed by Fixation and Area of Interest (*n = 2; 16.66%, each*). Heat Map, Entry Point, Pupil Dilation, Dwell Time, and Re-Fixation were each reported once (*n = 1; 8.3%*), whereas Scan Path and Gaze Path were not used or reported (*n = 0*). In relation to Balance, Saccade, and Gaze Path were the most utilized *(n = 4; 21.05% each*), followed by Fixation (*n = 3; 15.78%*). Area of Interest and Pupil Dilation were reported twice (*n = 2; 10.52% each*), while Heat Map, Entry Point, Dwell Time, and Re-Fixation appeared once (*n = 1; 5.26% each*). Scan Path was not used or reported (n = 0). For Gait, Saccade was again the most frequently used metric (*n = 4; 26.66%*), followed by Fixation (*n = 3; 20%*) and Gaze Path (*n = 2; 13.33%*). Heat Map, Area of Interest, Entry Point, Pupil Dilation, Dwell Time, and Re-Fixation appeared once (*n = 1; 6.66% each*) with Scan Path not used or reported (n = 0). Individual radar charts are available in the Supplementary Material section.

## 4 Discussion

### 4.1 Article bibliometrics

The reviewed studies demonstrate multiple authorships and span different publication years, reflecting the collaborative and evolving nature of research in this field, which is a pattern driven by the interdisciplinary demands of investigating how visual attention influences postural stability, as well as the growing global interest stemming from the rising proportion of older adults and associated public health concerns (Wood et al., 2022). Addressing postural stability in this population requires integrating expertise across geriatrics, neuroscience, physiotherapy (Wang J et al., 2025), and cognitive science (Szczepańska-Gieracha et al., 2016), naturally fostering multi-author collaborations supported by diverse institutions and funding bodies emphasizing importance of cross-disciplinary and international partnerships in addressing the multifactorial nature of falls among aging populations (Reed-Jones et al., 2013). This expanding multidisciplinary research within the network physiology of exercise, where visual attention strategies and postural stability intersect, reinforce the need for sustained collaborative efforts that integrate diverse methodological approaches and extend beyond controlled settings into real-world contexts. Moving forward, future studies should prioritize more inclusive populations, adopt longitudinal designs, and emphasize translational applications to better inform fall prevention strategies in the context of a rapidly aging population.

Reviewed studies were published in health-related journals despite reliance on technological tools and interventions, which suggests that the research remains clinically oriented, emphasizing outcomes such as fall prevention, balance improvement, and healthy aging among older adults. The technologies employed appear to serve primarily as supportive tools for healthcare practitioners in assessing network physiology of exercise, focusing visual attention, and postural stability (Bevilacqua et al., 2024) reflecting established norms in aging and rehabilitation research (Zeng et al., 2024), and the health sciences' dominance in interdisciplinary work (Davodi et al., 2023). This clinical focus, however, reveals a gap in interdisciplinary collaboration, where contributions from fields such as engineering, computer science, and human–computer interaction are still underrepresented. Greater integration across these fields is necessary to develop user-centered, scalable, and technologically robust interventions. Additionally, broadening dissemination in technology-focused and interdisciplinary journals could promote wider innovation and improve the real-world applicability of strategies to enhance balance and prevent falls among older adults.

The majority of reviewed studies recruited healthy participants with relatively small sample sizes, a pattern attributable to the methodological demands of simultaneously measuring two physiological systems under controlled conditions. Recruiting healthy participants minimizes confounding factors, such as comorbidities, medications, and age-related impairments (Mody et al., 2008), while specialized equipment and complex, time-intensive procedures often limits the number of participants that can be feasibly included, contributing to smaller sample sizes (Indrayan and Mishra, 2021); healthy participants are also better able to tolerate demanding protocols, enabling safer, more controlled data collection (Council for International Organizations of Medical Sciences CIOMS & World Health Organization, 2016). Although it suggests the existing research in this area remains largely preliminary, priortizing internal validity over broader applicability, this approach is valuable in establishing reliable baselines for understanding how the two systems interact under normal conditions before extending investigations to clinical populations. As such, findings may not generalize to older adults with co-existing conditions or functional limitations, who face a heightened risk of balance-related difficulties. Future studies should therefore recruit more diverse and clinically representative populations through multicenter or collaborative research designs and incorporate portable and wearable technologies to enable data collection in more naturalistic environments, thereby narrowing the gap between controlled experimental findings and real-world applications, particularly in developing effective assessment tools and interventions for fall prevention and rehabilitation among older adults.

The majority of reviewed studies recruited healthy participants with relatively small sample sizes, a pattern attributable to the methodological demands of simultaneously measuring two physiological systems under controlled conditions. Recruiting healthy participants minimizes confounding factors, such as comorbidities, medications, and age-related impairments (Mody et al., 2008), while specialized equipment and complex, time-intensive procedures often limits the number of participants that can be feasibly included, contributing to smaller sample sizes (Indrayan and Mishra, 2021); healthy participants are also better able to tolerate demanding protocols, enabling safer, more controlled data collection (Council for International Organizations of Medical Sciences CIOMS & World Health Organization, 2016). Although it suggests the existing research in this area remains largely preliminary, prioritizing internal validity over broader applicability, this approach is valuable in establishing reliable baselines for understanding how the two systems interact under normal conditions before extending investigations to clinical populations. As such, findings may not generalize to older adults with co-existing conditions or functional limitations, who face a heightened risk of balance-related difficulties. Future studies should therefore recruit more diverse and clinically representative populations through multicenter or collaborative research designs and incorporate portable and wearable technologies to enable data collection in more naturalistic environments, thereby narrowing the gap between controlled experimental findings and real-world applications, particularly in developing effective assessment tools and interventions for fall prevention and rehabilitation among older adults.

Most studies were completed in clinical or laboratory settings, where measurement tools such as force platforms, motion capture systems, and eye-tracking devices can be accurately deployed under stable conditions (Bonato et al., 2024), external variables minimized to more precisely isolate the relationship between visual attention and postural control (Ma et al., 2022), participant safety ensured through chlose supervision reducing the risk of falls among older participants (Appeadu and Bordoni, 2026), and standardized protocols applied to enhance the reliability and replicability of findings across studies (Committee on Reproducibility and Replicability in Science et al., 2019). Thereby, a controlled environment ensures the safety of respondents involving movement. Although laboratory-based studies enhance methodological rigor, they may fall short of capturing the dynamic and multifaceted conditions of daily life in which older adults navigate real-world environments. To advance the field, future research should adopt a combined methodology that integrates controlled laboratory experiments with assessments conducted in real-world or community-based settings. Incorporating portable and wearable devices, such as mobile eye-tracking systems and inertial sensors, can enable accurate and safe data collection beyond traditional laboratory settings. Expanding participant diversity to encompass frailer older adults or those with co-existing health conditions will further strengthen the generalizability of findings. Establishing uniform protocols across laboratory and field-based studies can enhance the reliability of results while increasing their relevance to clinical and community-based fall-prevention efforts.

The diverse and fairly even distribution across the reviewed study reflects the integrative nature of visual attention and postural stability, which involve interactions among cognitive, sensory, and motor systems and are therefore relevant across multiple health domains. These functions play important roles throughout the health continuum—from health promotion and fall prevention in healthy older adults to clinical assessment, treatment, and rehabilitation in at-risk populations (Balagué et al., 2020). As balance and fall risk are multifactorial and progressively change with aging, research naturally spans different stages and populations (Wang J et al., 2025), with technology serving as a flexible tool for assessment and intervention across various contexts (Kim et al., 2023). The physiological networks underlying exercise, particularly those governing visual attention and postural stability in older adults, are fundamentally multidisciplinary and relevant across all stages of life, underscoring the need for future studies employing longitudinal and integrative frameworks to monitor changes over time and across diverse health conditions. Researchers are encouraged to broaden their investigations to include diverse populations and real-world environments to strengthen the applicability of their findings, leverage technological advancements for personalized assessments and interventions, and promote collaboration across disciplines spanning geriatrics, neuroscience, physiotherapy, and technology development to advance both preventive and rehabilitative approaches.

The majority of the reviewed studies were conducted in the American Region, with limited representation from Southeast Asian, East Mediterranean, and European Regions. This geographic imbalance is notable given the extensive and aging body of exercise research in East Asia. For instance, a Japanese study (Shimada et al., 2017) reported that a 3-month aerobic, strength, and balance program increased brain glucose metabolism during walking in regions linked to spatial memory, alongside greater step length, which indicates enhanced gait control, postural stability, and visuospatial attention. Such Japanese research is largely absent from the current review, despite Japan’s strong gerontology base and rapidly aging population, primarily because of this review’s inclusion and exclusion criteria. This disparity may be largely driven by differences in research funding, infrastructure, and publication systems, as high-income countries tend to dominate aging research because they have stronger institutional support, larger research budgets, and better access to advanced technologies and specialized equipment for studying visual attention and postural stability (Jin et al., 2025) enabling more frequent and higher-quality research output (Li and Li, 2025). Thereby, supporting the factors contributing to the region’s global lead in older adults’ technology acceptance, further demonstrating its pace relative to other regions (Dino et al., 2025). In contrast, low- and middle-income regions often face financial and infrastructural constraints, as well as limited research capacity, which restrict their participation in highly specialized studies and reduce their representation in published literature (Dakhil et al., 2024). This outcome reflects significant global research inequality, suggesting that most existing knowledge is drawn from high-income populations and may not apply universally across diverse cultural or environmental settings. To address this issue, future efforts should prioritize studies in underrepresented areas by providing dedicated funding, strengthening local research capabilities, and fostering international partnerships. Cross-national and interdisciplinary approaches can bridge the gap between well-resourced and resource-limited regions, promoting greater diversity in both study populations and research methods. Building stronger research infrastructure and collaborative networks in low- and middle-income countries will foster a more balanced and globally inclusive understanding of visual attention and postural stability in aging populations, thereby enhancing the applicability and effectiveness of interventions across diverse settings.

The relatively high MMAT scores across the included papers are possibly due to the rigorous screening process that excluded studies with poor designs or incomplete data prior to the appraisal stage. Furthermore, the consistent use of validated eye-tracking technologies and standardized postural stability assessment tools, such as force platforms, the Timed Up and Go (TUG) test, and single-leg stance tests, directly contributed to their high ratings, as the MMAT framework recognizes the use of validated measurement instruments as a primary indicator of methodological quality. Furthermore, a critical comparison of the studies emphasized both convergence and heterogeneity. In context, there is consistent evidence identifying fixation and saccadic eye movements as primary visual attention metrics associated with postural stability. Moreover, studies with higher MMAT scores (80%–100%) consistently report that technology-assisted interventions, including virtual reality and gaze stabilization exercises, are effective in improving balance and reducing fall risk among older adults, supporting a moderate-to-high level of evidence. However, methodological and clinical heterogeneity remain evident. Variations in assessment approaches introduce differences in measurement precision and limit direct comparability across studies. Additionally, differences in study populations, particularly between healthy older adults and those with clinical conditions, further constrain the generalizability of findings.

### 4.2 Article purpose and outcome

The articles’ purposes and outcomes are organized into five themes, collectively referred to as the “5Ps of Visual Attention and Postural Stability.” Under “Performance”, vision and eye movement processes significantly influence postural stability. Visual input guides spatial orientation, and coordinated eye movements enhance balance, while disruptions in visual processing reduce it. Among older adults, visual input becomes increasingly critical as it compensates for age-related declines in proprioceptive and vestibular systems (Neumann et al., 2025; Wang Y et al., 2025; Wood et al., 2022). Structured interventions such as gaze stability exercises (GSE) strengthen the visual–vestibular system, improving dynamic balance, perception, and cognition (Mueangson et al., 2025), whereas reduced saccadic eye movement (SEM) speed in adults over 80 is associated with slower walking and poorer performance on measures like the Timed Up and Go (TUG) test (Bae, 2022). These findings underscore the value of incorporating visual and eye-movement training into routine balance assessment and rehabilitation to maintain postural stability and reduce fall risk.

Other than “Performance”, the “Program” domain shows that gaze-based and technology-assisted interventions were effective in improving stability and reducing fall risk. Wearable sensors and similar tools provide real-time, multisensory feedback—such as vibration, audio, or visual signals—that enables immediate postural adjustment, reducing sway and enhancing motor recovery (Duan et al., 2025; Kwon et al., 2024). Complementarily, structured and repetitive interventions such as vestibular rehabilitation therapy (VRT), task-oriented training, and gaze stabilization exercises promote neuroplasticity by repeatedly engaging the visual, vestibular, and motor systems together, thereby improving coordination, balance, gait, and sensory integration (Tramontano et al., 2025). Combining real-time feedback technologies with structured, repetitive training is recommended to maximize motor learning and reduce fall risk across populations.

Under “Process” cognitive and attentional demands were consistently associated with variations in balance performance. When attentional capacity is limited or cognitive load increases, the brain becomes less efficient at integrating visual, vestibular, and proprioceptive inputs. This is especially evident in older adults, whose postural control increasingly relies on attentional resources (Ge et al., 2021). In dual-task situations, divided attention often leads to reduced stability, and age-related declines in executive functions such as working memory and inhibition further compromise adaptive balance responses (Uiga et al., 2020). These findings may suggest that balance assessment and rehabilitation should incorporate dual-task exercises combining cognitive and postural challenges to better reflect real-life conditions.

Within the “Product” domain, standardized assessment tools proved effective at capturing the integrated relationship among balance, gaze, and cognitive functions. By using validated, comparable metrics across individuals and studies, these tools allow different functional domains to be assessed within a unified framework. This highlights that isolating any single domain may overlook important interactions between systems. Clinicians and researchers are therefore encouraged to prioritize multi-domain or standardized tools when evaluating postural control and fall risk in older adults.

Finally, the “Person” theme reveals that age and clinical conditions are linked to reduced postural stability, reflecting declines in both sensory and cognitive systems. Reduced visual acuity and slower visual processing hinder spatial orientation (Wang Y et al., 2025), while diminished attentional capacity and processing speed limit older adults' ability to manage balance during complex or dual-task situations such as walking while talking (Ercan Yildiz et al., 2024). As postural control becomes less automatic with aging and disease, maintaining balance demands greater conscious effort, increasing the risk of dual-task interference and falls. Balance interventions for these populations may incorporate dual-task conditions and combine motor training with cognitive exercises to enhance attentional capacity and reduce fall risk.

### 4.3 Keyword network analysis

Keyword network analysis of articles related to older adults’ visual attention and postural stability showed that research is organized into thematically clusters, topics: “Vision”, “Virtual”, “Vulnerability”, “Visual-Motor”, “Velocity”, “Vestibular”, and “Vergence”. The clusters “Vision”, “Virtual”, and “Visual-Motor” clusters reflect studies on interactive, technology-based platforms, including video games and virtual reality, designed to enhance visual attention, visuomotor coordination, and functional mobility. The “Vulnerability”, “Vestibular”, and “Vergence” clusters highlight the impact of age-related, clinical, and cognitive-perceptual factors on postural stability and balance impairments. Finally, the “Velocity” cluster emphasizes the role of visual motion cues, such as optical flow, in supporting postural control and fall prevention. The clustering of the first three categories underscores the growing use of video games and virtual reality for training visual attention and mobility, suggesting that interactive, technology-driven programs can be effective tools to enhance balance and functional movement in older adults (Ciemer et al., 2025). This also reflects a paradigm shift toward digital and interactive rehabilitation, where activities provide customizable, engaging, repeatable, and feedback-driven environments that enhance visual attention and motor learning (Georgiev et al., 2021). It is recommended that platforms be developed to adjust difficulty levels, task complexity, and feedback in real time to match individual capabilities, maximizing training effectiveness and safety.

The “Vulnerability”, “Vestibular”, and “Vergence” clusters highlight that balance impairments are strongly influenced by age, comorbidities, and cognitive-perceptual challenges. This may indicate the interaction among sensory decline, cognitive load, and underlying clinical conditions rather than a single deficit among individuals (Whitson et al., 2018). Similarly, the presence of vestibular- and vergence-related keywords supports the idea that sensory integration, particularly visual-vestibular interaction, is critical for maintaining balance, and disruptions in these systems significantly increase fall risk (Chaudhary et al., 2022). Assessment of postural stability should therefore go beyond basic balance tests to include evaluation of visual, vestibular, and cognitive-perceptual functions, ensuring a more comprehensive understanding of fall risk. Lastly, keyword clustering under “Velocity” emphasizes the role of optical flow and other visual motion information in postural control, highlighting that accurate perception of environmental movement is essential for postural regulation, especially during dynamic tasks such as walking (Salinas et al., 2017). To improve perception of environmental movement and postural adaptation, it will be helpful to incorporate dynamic visual motion stimuli into balance programs, especially among older adults. Further, the sparse number of studies suggests that the keyword network analysis serves as an initial conceptual map rather than an exhaustive or final depiction of the discipline.

### 4.4 Technology-driven assessment tools and metrics measuring various attributes of visual attention and postural stability

The assessment of visual attention in the articles is heavily shaped by technology-based measurement tools, with fixation metrics receiving the most attention, followed by saccades. The prominence of eye-tracking tools for these metrics may stem from the combination of theoretical and physiological perspectives, as well as from the capabilities of existing measurement technologies. Fixations and saccades are the fundamental means of interacting with and perceiving the visual environment (Mahanama et al., 2022). This is supported by the foundational eye-tracking methodology (Duchowski, 2017; Holmqvist et al., 2011), which reported that these two metrics, along with smooth pursuit, are sufficient to model the overt localization of visual attention. Fixation occurs when the eye is stationary and locked on a specific point to process information, while the rapid movements between fixations are called saccades (Al-Moteri et al., 2017). These behaviors are rooted in the functional organization of the visual system, giving them properties such as low spatial dispersion, distinct velocity values, and ballistic movement. One of the most fundamental properties that enable the detection of fixations is low spatial dispersion: the gaze remains clustered within a small area of the visual field, producing low velocity values that contrast sharply with saccadic movements (Lobão-Neto et al., 2022). Similarly, saccades are rapid ballistic movements that redirect the fovea toward objects of interest, characterized by high velocity and a predictable relationship among amplitude, duration, and peak velocity (Gibaldi and Sabatini, 2021), a property that further anchors their reliable identification in eye-tracking systems. Having these characteristics made them the most readily detectable and quantifiable oculomotor behaviors with the current eye-tracking technology. Overall, research on visual attention metrics using technology-based eye-tracking suggests that they are largely shaped by what current systems can reliably measure, rather than by the full complexity of how visual attention actually works (Jayawardena et al., 2024). This results in an overrepresentation of simpler oculomotor metrics, while more complex processes remain understudied, creating a gap that presents opportunities for future studies. It requires going beyond fundamental metrics to reveal deeper insights into visual attention systems by investing in more advanced eye-tracking technologies that can capture a wider, more complex range of visual attention behaviors.

The dominance of fixation and saccade metrics is further reflected in the limited use of other technology-driven visual attention measures, such as pupil dilation, re-fixation, heat maps, and entry points. This limitation appears to stem from both analytical complexity and data processing demands, leading developers to prioritize metrics that are more directly and reliably extracted from raw eye-tracking signals. To clarify this distinction, unconscious eye-movement indicators (e.g., pupil dilation and blink rate) are differentiated from conscious metrics such as fixations and saccades (Liang et al., 2025). This distinction is important, as conscious measures show stronger associations with performance outcomes, whereas pupil dilation suggests weaker and less consistent predictive value for situation awareness, limiting its use as a primary indicator. Similarly, heat maps are typically derived visualizations rather than independent data sources, constructed from fixation distributions rather than representing raw oculomotor events. This dependency is demonstrated by reconstructing heat maps back into fixation points (Othman et al., 2020) with heat maps further described as secondary outputs generated from gaze data (Gnanaraj et al., 2025). Re-fixation is likewise treated as a derivative measure embedded within broader fixation duration and frequency analyses (Sousa et al., 2025), while entry point metrics remain underrepresented in the literature, likely due to their limited methodological standardization and narrower applicability. Collectively, these patterns suggest that the prominence of fixation and saccade measures is driven not only by theoretical relevance but also by practical and technological constraints, as developers tend to rely on metrics that are most reliably extracted from eye-tracking systems. This reliance produces a partial representation of visual attention, where potentially informative but more complex metrics—particularly pupil dilation and heat-based representations—remain underutilized due to processing demands rather than theoretical insignificance, underscoring the need to reassess their analytical value in future research.

Notably, the results revealed that no documented technology-driven assessment tool exists to measure scan path. Among the given visual attention metrics, scan path appears to be the most complex to quantify, as it captures both spatial organization and temporal dynamics of gaze (Laborde et al., 2026), and this complexity has yet to be simplified into a clinically interpretable metric. Recent learning-based scan path models still face issues, particularly spatial misalignment of fixation features and underutilization of temporal dynamics, thereby constraining their effectiveness in downstream task representation (Zhong et al., 2024). Furthermore, highlight the lack of a single visualization method that could convey both spatial and temporal aspects of eye movement simultaneously. For instance, the head-mounted eye-tracking device evaluated in the study of Diaz, (2023) demonstrated a trade-off: it either improves spatial accuracy by increasing spatial resolution or increases temporal sampling rate—never both. This implies that the field remains fragmented, with diverse representations and metrics that complicate interpretation and method selection (Laborde et al., 2026), and that reducing these to a single clinically actionable metric remains challenging. To address this, future work should focus on developing a standardized protocol and integrating multiple complementary metrics to make data easier to compare and apply in clinical practice. In addition, scan path analysis provides a nuanced window into the dynamics of visual attention despite the complexity and measurement challenges. Consequently, integrating scan path metrics alongside established measures provides future research with a deeper understanding of attentional mechanisms and offers complementary mechanistic insights not captured by traditional metrics alone.

There are numerous technology-driven assessment tools and metrics used to evaluate posture and balance under the broader domain of postural stability. These range from conventional clinical movement scales to more technology-enhanced systems, often combining simple, low-cost tools with clinical judgment to produce non-invasive, accessible, and immediate assessments. Such approaches are well-established and widely implemented in both clinical and research settings, reflecting a gradual shift away from purely traditional assessment methods. Although conventional tools were once the primary means of evaluation, they have since been complemented—and in many contexts superseded—by more advanced techniques, although they remain in use when technological resources are limited (Veqar, 2014). Postural and balance assessments are recognized as critical indicators of health status and essential tools for the early detection of balance impairments (Noamani et al., 2023; Sugiyama et al., 2024). Furthermore, because functional independence in daily activities depends heavily on intact balance control, older adults are particularly vulnerable to its decline, which increases fall risk and reliance on assistance as age and illness progress (Nnodim, 2015). Accordingly, postural and balance assessments remain dominant within research and clinical practice involving older adults. Future development should focus on modernizing traditional assessment approaches through deeper technological integration, enabling a shift toward more objective, high-precision metrics for evaluating posture and balance. However, such advancement should be approached cautiously to ensure that data-driven measures complement rather than replace the clinical judgment and holistic observation that remain essential in geriatric care.

There are five technology-driven assessment tools, such as the one-legged stance test, stabilometer, inclinometer, force platform posturography technique, and functional reach (FR) test and timed up and go (TUG), that measure multiple postural stability metrics, including posture, stability, and balance. These are used alongside technologies to assess these categories. These scales have long been established in clinical and research settings, demonstrating their reliability and validity in measuring multiple metrics of postural stability. Moreover, these scales assess functional and real-world performance, which can be paired with technology to achieve comprehensive measurement coverage. Previous studies confirm that instruments such as the functional reach (FR) test, one-legged stance test, or single-leg stance (SLS), timed up and go (TUG) are valid, reliable, and crucial for identifying certain balance problems (Albalwi et al., 2025; Nakhostin-Ansari et al., 2022). To provide a thorough assessment of postural instability and gait disorders, objective clinical examinations are combined with simple equipment, making the assessment quick to perform (Mak, 2021) and consistent with modern practice (Mak, 2021). Overall, the results indicate the use of both traditional methods and technology-driven assessment tools to measure posture, stability, and balance. It is suggested that future research must integrate artificial intelligence (AI) driven tools. These may include machine learning (ML) algorithms, predictive analytics, and assistive robotics, enabling enhanced disease detection, clinical decision-making, and proactive monitoring, thereby revolutionizing geriatric care (Abadir et al., 2025; Tana et al., 2025). While the integration of AI and ML, which can produce algorithms, analytics, and assistive robotics, offers transformative potential for geriatric care and postural stability assessment, it is important for researchers to ensure these technologies maintain the clinical validity and ethical reliability required for clinical decision-making.

Gait has the fewest technology-driven assessment tools within postural stability research. This scarcity likely reflects both technological limitations in gait measurement and the continued reliance on clinical tests and standardized scales. Existing literature indicates that gait analysis remains underutilized beyond clinical contexts such as orthopedic, neurological, and surgical examinations, despite its potential utility in prevention and health promotion by identifying early functional decline before progression to disease (Roggio et al., 2021). Traditional gait assessment methods, including force plates and three-dimensional motion capture systems, are also constrained by high cost, specialized training requirements, and restricted use in controlled laboratory settings (Kumar et al., 2025). These constraints contribute to the limited number of technology-driven gait assessment tools and the continued dominance of observational tests and rating scales. This gap underscores the need to expand technology-enabled approaches for gait evaluation beyond clinical examination settings. Recent developments in wearable technologies offer promising alternatives, with devices such as accelerometers, activity monitors, and pressure sensors enabling noninvasive, real-time measurement of gait parameters including cadence, stride length, gait cycle duration, and symmetry (Kumar et al., 2025; Sakellari and Gioftsos, 2025). These advances suggest a growing opportunity for researchers and developers to further refine and standardize wearable-based gait analysis tools to complement or extend current assessment approaches.

### 4.5 Visual attention metrics in the context of postural stability

Across posture, stability, balance, and gait-related studies, saccades emerged as the most consistently reported visual attention metric, reflecting a pervasive preference in the literature for measuring rapid eye movements across all four task categories. In posture-related studies, fixation was as prominent as saccades, suggesting that researchers investigating postural control capture both discrete eye-movement events and periods of stable gaze. In balance-related studies, gaze path appeared alongside saccade as a frequently reported metric, indicating a broader characterization of visual attention that extends beyond discrete movements to encompass continuous gaze trajectories. By contrast, studies on stability and gait placed greater emphasis on saccades, reflecting a stronger and more uniform preference for rapid eye movement measures in locomotor and stability contexts.

Saccades predominate across all categories because they directly index proactive visual sampling, a process relevant to any motor control task requiring environmental scanning (Henin et al., 2025). Because posture, stability, balance, and gait all demand continuous environmental monitoring, researchers consistently favor saccades as the primary metric for capturing how the visual system actively gathers information to support motor control. This predominance of saccades across stability and gait studies, alongside the broader range of visual attention measures observed in posture and balance research, is also consistent with the previous network physiology framework, which proposes that physiological systems dynamically adjust the organization of their interactions depending on task demands. Fixation co-dominates postural studies because visual-postural coupling inherently demands gaze stabilization alongside rapid visual shifts, as continuous gaze adjustments compensate for body motion to maintain postural stability (Lafleur and Lajoie, 2023; Park et al., 2024; Wibble and Pansell, 2024). Gaze path is a prominent area of research because the vestibular system continuously monitors head movement and transmits real-time signals to stabilize gaze during motion (Boyle, 2021). As the vestibular system provides constant sensorimotor feedback about body position, gaze behavior reflects an ongoing dynamic process rather than isolated static moments. Gaze path, as a continuous trajectory measure, captures this feedback loop over time, making it a more sensitive indicator of moment-to-moment balance adaptation than discrete metrics (Treger et al., 2020). In stability and gait studies, saccades alone dominate as the preferred metric for visual attention, consistent with the established functional link between saccadic behavior and locomotor performance. This relationship is evidenced by a positive correlation between saccade duration and gait velocity in individuals with mTBI, with reduced saccade duration associated with slower walking speed (Lirani-Silva et al., 2021).

The use of visual attention metrics in postural stability research among older adults suggests that the visual-postural relationship is fundamentally mediated by interconnected physiological networks rather than isolated sensory mechanisms. From a network physiology perspective, proprioceptive, visual, vestibular, and cognitive systems function as an integrated network that regulates postural stability (Manzari et al., 2022). Posture, balance, and gait serve as fundamental determinants of human movement, independence, and quality of life, each relying on the coordinated interaction of musculoskeletal, sensory, and neural systems (Sakellari and Gioftsos, 2025). Sensory Integration Theory (SIT) further supports this network physiology framework. SIT identifies tactile, proprioceptive, and vestibular input as the three primary sensory systems underlying postural control, bilateral integration, and praxis (Alotaibi et al., 2025). Central to this network is the postural control system, which maintains balance against gravity and orients body segments as a reference frame for perception and action. This system operates through four components: internal representations of body orientation and center of gravity, multisensory inputs regulating body segment stabilization, and flexible postural reactions or anticipatory adjustments for balance recovery and voluntary movement stabilization (Massion, 1994). Among older adults, age-related neurodegeneration disrupts the integrity of these inter-system connections, reducing the efficiency of multisensory integration required for gaze stabilization and rapid visual shifts during postural adjustments (Pepper and Nuttall, 2023). Interpreting these findings through the lens of network physiology advances the discussion beyond isolated visual metrics, positioning eye-tracking technology as a critical network-level indicator of postural stability. This perspective carries significant implications for developing targeted diagnostic tools and neurorehabilitation interventions for the older adult population.

Future studies are recommended to further explore the application of eye-tracking technology in postural research among older adults, particularly within the framework of network physiology. Emphasis should be placed on establishing the acceptance, reliability, and standardization of eye-tracking metrics. Standardizing these metrics would not only strengthen the methodological rigor of visual-postural research but also facilitate its integration into broader sensorimotor network assessments. Moreover, validated and standardized eye-tracking protocols would support the development of reliable diagnostic tools to detect early disruptions in the visual-postural network, as well as preventive frameworks to mitigate fall risk and postural decline in the older adult population.

### 4.6 Novel contributions and implications

The findings of this review support the integration of visual attention into routine physiotherapy assessment of postural stability among older adults. Evidence indicates that visual attention is closely associated with balance, gait, and functional performance (Alghamdi et al., 2021), while age-related impairments such as delayed eye–body coordination and reduced postural control further contribute to fall risk (Cinelli et al., 2008; Rodrigues et al., 2023). In addition, the presence of combined vestibular, visual, and motor deficits highlights the need for a multidimensional assessment approach (Tuunainen et al., 2011). Clinically, this may suggest that physiotherapists should incorporate visually demanding and dual-task conditions into balance assessments to better reflect real-world functional challenges and identify deficits that may not be captured through conventional testing alone.

Eye-tracking technology offers a practical and objective method to operationalize this approach in clinical settings. It enables the quantification of gaze behavior, including fixation patterns, visual attention allocation, and eye–body coordination during postural tasks (Maldonado-Díaz et al., 2025). Such metrics can be used to (1) identify abnormal gaze strategies associated with instability, (2) monitor changes in attentional control during rehabilitation, and (3) evaluate patient performance under dual-task conditions. The demonstrated reliability of platforms measuring gaze, balance, and cognition further supports their clinical applicability for tracking patient progress over time (Szturm et al., 2015). When available, integrating eye-tracking into assessment protocols may enhance clinical decision-making, particularly in older adults with cognitive impairment, dizziness, or neurological conditions.

From an intervention perspective, the evidence supports the incorporation of gaze-oriented strategies into physiotherapy programs. Gaze stability exercises combined with balance training have been shown to improve postural control and reduce fall risk in older adults with dizziness and related conditions (Fatima et al., 2022; Mitsutake et al., 2018). Visual-guided postural training and vergence eye movement exercises also contribute to improved stability and visual–motor integration (Chen et al., 2025; Mitsutake et al., 2018). Eye-tracking can further be used during intervention to provide real-time or post-session feedback on gaze behavior, allowing clinicians to tailor exercises based on individual attentional patterns and to ensure appropriate visual engagement during tasks. Multicomponent programs that integrate visual, cognitive, and motor elements—such as stepping exercises with gaze demands—appear particularly effective in improving functional outcomes and reducing falls (Yamada et al., 2013).

Technology-assisted rehabilitation, including game-based and virtual reality platforms, provides an additional avenue for applying these principles in practice. Performance in visually demanding tasks has been shown to reflect underlying visual attention and balance abilities (Alghamdi et al., 2021), while virtual environments combined with eye-tracking can facilitate simultaneous training of cognitive–motor interactions (Maldonado-Díaz et al., 2025). However, inconsistent findings from isolated visual attention training interventions suggest that these approaches should be embedded within comprehensive, multicomponent rehabilitation programs rather than used alone (Althomali et al., 2019).

Moreover, the identification of specific visual factors associated with impaired balance emphasizes the need to extend clinical evaluation beyond standard vision screening (Althomali and Leat, 2018). Vestibular rehabilitation, although underutilized, also indicates potential in addressing balance impairments across neurological populations (Abasi et al., 2022). While further research is warranted to strengthen the evidence base, current findings support the clinical integration of visual attention assessment and gaze-based interventions—including the use of eye-tracking where feasible—as part of a comprehensive strategy to improve postural stability and reduce fall risk in older adults.

### 4.7 Limitations

This review is firstly limited by the heterogeneity of the included studies across populations, methodologies, and outcome measures. Participant samples varied from healthy older adults to those at higher risk of falls, with differences in age and physical activity levels, while visual attention was assessed using different eye-tracking systems and metrics such as fixation, saccades, and gaze behavior. Postural stability was likewise measured using a range of approaches, including force platforms, gait analyses, and clinical balance scales, often under varying task conditions such as static, dynamic, and dual-task paradigms. This diversity reflects the breadth of approaches in the field but also represents a limitation in terms of comparability across studies.

An additional limitation of this review is the absence of a quantitative synthesis. Although such an approach could provide stronger estimates of the association between visual attention and postural stability, it was not feasible due to substantial heterogeneity in outcome measures, visual attention metrics, and study designs. Several included studies did not report sufficient statistical data (e.g., effect sizes, correlation coefficients, or convertible statistics such as t- or F-values), limiting the ability to derive comparable estimates. Future research would benefit from more standardized reporting of statistical outcomes to enable meta-analytic synthesis.

While this review followed strict guidelines, its scope is limited by a small sample size of only 15 studies. This makes the mapping and network analyses less definitive than usual. The evidence base included in this review also presents several limitations that should be considered when interpreting the findings. First, the included studies generally involved small sample sizes (mean = 48 participants), which may limit the statistical power and generalizability of results. Second, a substantial proportion of studies (66%) were conducted in controlled laboratory settings, which, while useful for isolating specific variables, may not fully capture real-world conditions where postural control and visual attention operate in more complex and dynamic environments. Third, there is a notable geographic concentration of studies, with 47% conducted in the Americas, potentially limiting the diversity of populations and contextual factors represented in the literature. These limitations highlight the need for future research with larger, more diverse samples and increased use of ecologically valid designs to strengthen the applicability of findings.

## 5 Conclusion

This systematic review synthesized evidence on the relationship between visual attention and postural stability among older adults participating in health-enhancing physical activity, framed through a network physiology perspective that treats physiological systems as dynamically interconnected rather than operating in isolation. Across bibliometric, thematic, and network dimensions, a coherent picture emerges: visual attention is a central, modifiable determinant of postural control that warrants deliberate integration into both assessment and intervention frameworks.

In bibliometric analysis, research output peaked in 2022, with the Americas contributing the largest share of studies. Most were conducted in laboratory and clinical settings with small, predominantly healthy samples, limiting generalizability to community-dwelling and clinically complex populations. Thematically, the literature organized around five interrelated domains—the “5Ps”: Performance, Program, Process, Product, and Person—reflecting the multidimensional nature of visual–postural interaction consistent with a network physiology view of balance as an emergent property of interconnected sensory, cognitive, and motor systems. Keyword network analysis further confirmed that the field conceptually recognizes this complexity, mapping themes across virtual reality–based interventions, aging-related vulnerabilities, visuomotor coordination, vestibular function, and dual-task challenges—each representing a node in the broader physiological network underlying postural control. From a measurement standpoint, fixation and saccade metrics dominated, while advanced indicators such as heat maps, pupil dilation, dwell time, and re-fixation patterns remained underutilized. This reliance on basic oculomotor metrics may obscure richer attentional dynamics relevant to postural stability under real-world conditions. The heterogeneity of platforms, task conditions, and outcome definitions across studies also limits cross-study comparability—a constraint that must be acknowledged when interpreting cumulative findings.

Furthermore, the evidence supports incorporating visually demanding and dual-task conditions into routine physiotherapy assessment, as conventional balance tests may fail to detect attentional deficits relevant to fall risk. Through a network physiology lens, these deficits reflect disruptions in the coordinated interplay among visual, vestibular, cognitive, and motor networks rather than failures of any single system. Where accessible, eye-tracking offers an objective means of quantifying gaze behavior and monitoring attentional changes across rehabilitation. Gaze stability training combined with balance exercises, visual-guided postural programs, and multicomponent interventions integrating visual, cognitive, and motor demands—including game-based and virtual reality formats—appear most effective precisely because they simultaneously engage multiple physiological networks. Isolated visual attention training, however, should not be used as a standalone approach.

These conclusions are qualified by methodological limitations, including restriction to three databases and English-language peer-reviewed articles, exclusion of grey literature, and the absence of granular eye-movement descriptors in the search strategy. Population, tool, and paradigm heterogeneity precluded meta-analytic synthesis. Future research should expand the use of advanced visual metrics, recruit more diverse and clinically representative samples, standardize eye-tracking protocols, and evaluate long-term functional outcomes beyond laboratory settings—ideally adopting network physiology frameworks that can model the complex, nonlinear interactions among physiological systems.

Overall, this review supports a network physiology model of postural stability in which visual attention, vestibular function, cognitive processing, and motor output are dynamically interconnected nodes of an integrated system. Translating this understanding into practice requires assessment and intervention approaches that embrace gaze-based strategies, technology-assisted platforms, and multicomponent program designs as part of evidence-based, person-centered fall prevention.

## Data Availability

The data presented in the study are deposited in the FigShare repository, accession number: https://doi.org/10.6084/m9.figshare.32562567.
