# Supplementary material for: Visual attention and postural stability among older adults participating in health-enhancing physical activity: a systematic review
Source: Front Netw Physiol. 2026 Jun 8;6:1841735. doi: 10.3389/fnetp.2026.1841735 (PMC13283787; doi:10.3389/fnetp.2026.1841735)

**Supplementary file:**

Supplementary File: Individual Article Purpose and Outcome

| No. | Research | QC^A^ | R^B^ | Purpose | Outcomes |
| --- | --- | --- | --- | --- | --- |
| 1 | Abasi et al., 2022 | 80% | 3 | To examine the effects of vestibular rehabilitation on oculomotor function. | Vestibular rehabilitation is underutilized but can improve balance and address impairments in individuals with neurological conditions. |
| 2 | Alghamdi et al., 2021 | 80% | 4 | To investigate the relationship between visual attention, balance, mobility, and performance in Kinect games. | Visual attention measures are linked to performance in Kinect games and are also associated with balance and gait abilities. |
| 3 | Chen et al., 2025 | 80% | 5 | To examine how short-term interactive balance training influences cortical reorganization in older adults. | Short-term visual-guided postural training improved stability in older adults, alongside enhanced brain activity and network organization. |
| 4 | Cinelli et al., 2008 | 60% | 4 | To compare balance and body segment control between older and younger adults during gaze reorientation tasks. | Older adults showed delayed coordination between eye movements and body rotation, indicating age-related physiological decline. |
| 5 | Souza et al., 2022 | 80% | 5 | To examine the eye–posture relationship in older fallers, focusing on visual stabilization mechanisms. | Older adults with a history of fall had poorer balance and eye movements than nonfallers, though their eye–posture relationship remained functional. |
| 6 | Fatima et al., 2022 | 80% | 4 | To compare balance training with and without gaze stabilization exercises in elderly patients with chronic dizziness. | Combining gaze stability and balance exercises effectively improves balance and reduces fall risk in older adults with dizziness. |
| 7 | Maldonado-Diaz et al., 2025 | 80% | 5 | To investigate visual attention using eye-tracking during VR-based balance training in older adults with cognitive impairment. | Eye-tracking provides useful insights into attentional behavior during balance training and supports VR-based rehabilitation approaches. |
| 8 | Matheron et al., 2016 | 100% | 5 | To assess the effects of vision, vergence, viewing distance, and cognitive load on postural control in older adults. | Postural stability decreased with far focus and eye closure but improved with vergence, highlighting proximity-related benefits in older adults. |
| 9 | Mitsutake et al., 2017 | 100% | 5 | To investigate the effects of posterior circulation stroke (PCS) on postural stability and evaluate gaze stability exercises in PCS and non-PCS groups. | Gaze stability exercises improve postural control in PCS patients, particularly during dynamic standing tasks. |
| 10 | Rodrigues et al., 2023 | 100% | 5 | To examine postural control in diabetic older women during fixation and horizontal eye movements. | The diabetic group showed poorer postural control, reflected by increased sway amplitude and velocity. |
| 11 | Szturm et al., 2014 | 80% | 5 | To evaluate the reliability and validity of a dual-task platform for assessing balance, gaze, and cognition in older adults. | The platform showed moderate to high reliability and consistently detected changes in balance, gaze, and cognition under dual-task conditions. |
| 12 | Tuunaeinen et al., 2010 | 100% | 5 | To examine the relationship between vestibular symptoms and objective vestibular, oculomotor, and balance measures in older adults. | Most elderly participants exhibited vestibular and balance impairments, with reduced postural control and multiple causes of dizziness and falls. |
| 13 | Yamada et al., 2013 | 100% | 5 | To assess the effects of a multitarget stepping program with multicomponent exercise on stepping, gaze, and fall outcomes. | A twice-weekly multitarget stepping program combined with multicomponent exercise reduced falls and fractures while improving stepping, gaze, and physical performance over 12 months. |
| 14 | Althomali et al., 2019 | 100% | 5 | To determine if visual attention training can improve balance and mobility among older adults. | Visual attention training showed no improvements in balance or mobility among older adults. |
| 15 | Althomali and Leat, 2018 | 100% | 5 | To determine the relationship of balance, mobility, and fear of falling, and aspect of vision. | Multiple visual factors contribute to impaired balance and mobility beyond what standard vision tests capture. |

QC^A^ = Quality Check: 0-100% (0 - poor; 100 - excellent); R^B^ = Relevance: 0-5 (0 - not relevant; 5 - highly relevant)

Supplementary File: Individual Radar Charts for Visual Attention and Postural Stability Measurements
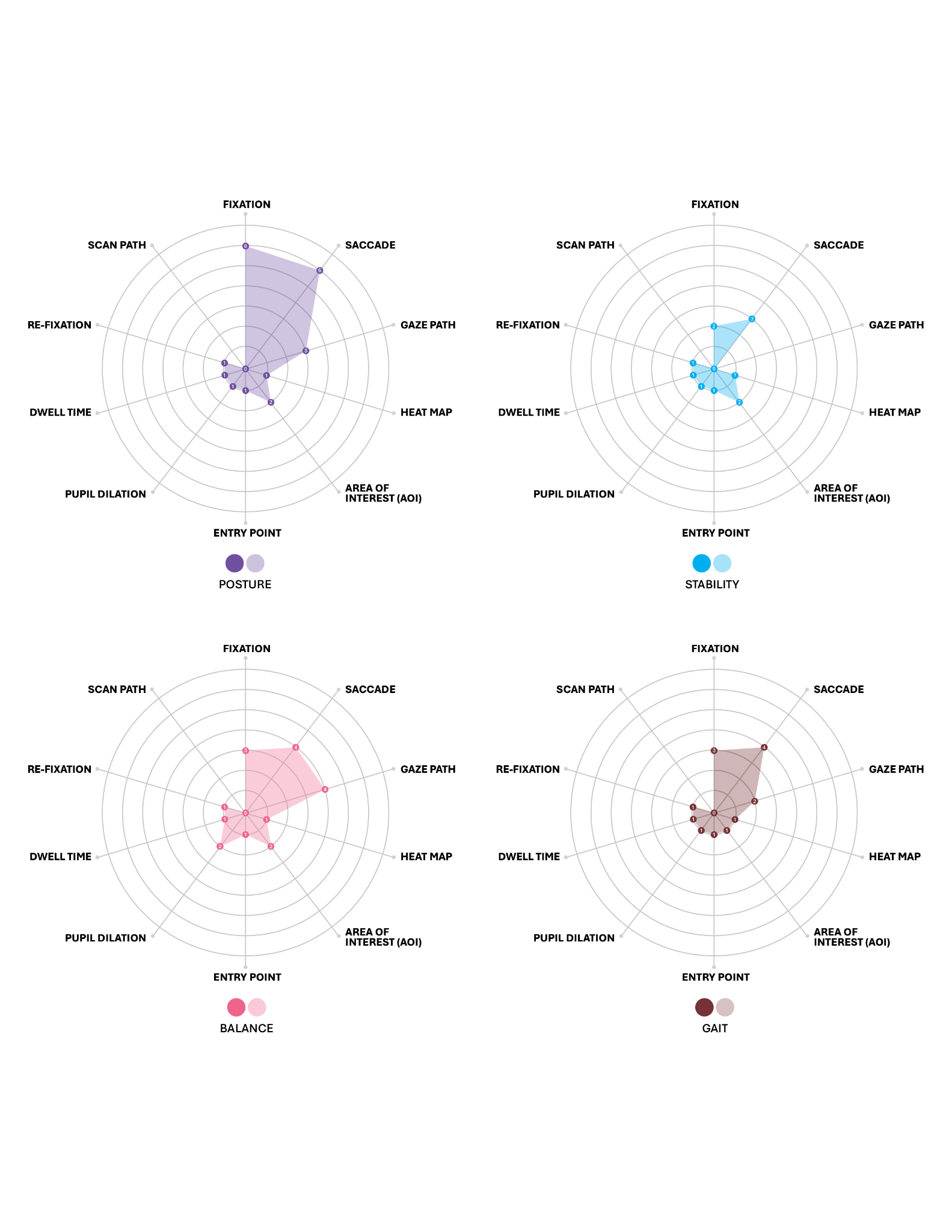

Supplement: Supplementary file 2 [file Table2.docx]
